# Direct Interaction of Selenoprotein R with Clusterin and Its Possible Role in Alzheimer’s Disease

**DOI:** 10.1371/journal.pone.0066384

**Published:** 2013-06-21

**Authors:** Ping Chen, Chao Wang, Xiaojie Ma, Yizhe Zhang, Qing Liu, Shi Qiu, Qiong Liu, Jing Tian, Jiazuan Ni

**Affiliations:** 1 Changchun Institute of Applied Chemistry, Chinese Academy of Sciences, Changchun, P.R. China; 2 College of life Sciences, Shenzhen Key Laboratory of Microbial Genetic Engineering, Shenzhen University, Shenzhen, China; 3 University of Chinese Academy of Sciences, Chinese Academy of Sciences, Beijing, P.R. China; 4 College of Optoelectronic Engineering, Shenzhen University, Shenzhen, P.R. China; Cleveland Clinic Foundation, United States of America

## Abstract

Selenoprotein R (SelR) plays an important role in maintaining intracellular redox balance by reducing the R-form of methionine sulfoxide to methionine. As SelR is highly expressed in brain and closely related to Alzheimer′s disease (AD), its biological functions in human brain become a research focus. In this paper, the selenocysteine-coding TGA of *SelR* gene was mutated to cysteine-coding TGC and used to screen the human fetal brain cDNA library with a yeast two-hybrid system. Our results demonstrated that SelR interacts with clusterin (Clu), a chaperone protein. This protein interaction was further verified by fluorescence resonance energy transfer (FRET), coimmunoprecipitation (co-IP), and pull-down assays. The interacting domain of Clu was determined by co-IP to be a dynamic, molten globule structure spanning amino acids 315 to 381 with an amphipathic-helix. The interacting domain of SelR was investigated by gene manipulation, ligand replacement, protein over-expression, and enzyme activity measurement to be a tetrahedral complex consisting of a zinc ion binding with four Cys residues. Study on the mutual effect of SelR and Clu showed synergic property between the two proteins. Cell transfection with SelR gene increased the expression of Clu, while cell transfection with Clu promoted the enzyme activity of SelR. Co-overexpression of SelR and Clu in N2aSW cells, an AD model cell line, significantly decreased the level of intracellular reactive oxygen species. Furthermore, FRET and co-IP assays demonstrated that Clu interacted with β-amyloid peptide, a pathological protein of AD, which suggested a potential effect of SelR and Aβ with the aid of Clu. The interaction between SelR and Clu provides a novel avenue for further study on the mechanism of SelR in AD prevention.

## Introduction

Selenium (Se) exerts its biological function mainly through selenoproteins in which Se is present in the form of selenocysteine (Sec) that is encoded by a traditional stop codon (TGA) in the open reading frame [1]–[3]. The dual functions of TGA codons result in low efficiency of selenoprotein expression, which makes studying their structure and function difficult [4]. Selenoprotein R (SelR, also called methionine sulfoxide reductase B1 (MsrB1)) was first identified as a selenoprotein through bioinformatics methods [4]. SelR can be found in both the cytoplasm and nucleus of mammalian cells. It can stereospecifically catalyze the reduction of oxidized methionine (i.e., methionine sulfoxide to methionine (Met) residue in proteins [5], [6]. Two other non-Se-containing MsrB enzymes (MsrB2 and MsrB3) comprise cysteine (Cys) at the active site and they are located in the mitochondria and endoplasmic reticulum [7].

Met oxidation is usually accompanied by an increase of intracellular ROS, which can damage proteins if sulfoxide is not reduced to Met by Msr catalysis [8]. Recent studies have shown that SelR is relevant to the lens cell survival, and silencing *SelR* can increase oxidative stress that causes lens cell death. This indicates that SelR plays a key role in conferring oxidative stress resistance and possibly preventing cataract formation [9]. Msr overexpression leads to increased ability to resist oxidation and to prolonged lifespan [10]. Se has been proposed to play a role in preventing Alzheimer′s disease (AD) [11]. As SelR is highly expressed in the brain [12], it may have anti-aging properties and neuronal protective functions. Thus it is very important to identify proteins that interact with SelR in brain, to explore SelR-mediated pathways, and to elucidate the biological function of SeR.

To date, only two proteins have been reported to interact with SelR. One is the transient receptor potential melastatin type 6 (TRPM6), with which SelR interacts to recover TRPM6 channel activity by reducing Met^1755^ oxidation and modulates TRPM6 during oxidative stress [13]. The other is Trx, whose interaction with SelR was verified by nuclear magnetic resonance [14]. In this study, SelR was found to interact with the chaperone protein clusterin (Clu) using yeast two-hybrid screening of a human fetal brain cDNA library. Further experiments demonstrated that the interacting domains were in the central region of Clu (aa 315–381) and the zinc tetrahedral structure of SelR. The mutual effect of SelR and Clu showed cooperation between the two proteins: SelR overexpression could increase Clu protein levels, and greater amounts of Clu increased SelR activity. The protein interaction could also increase SelR enzymatic activity and reduce intracellular ROS in an AD model cell line, N2aSW. As a mutation in the *Clu* gene was recently linked with AD [15], [16], the results described here imply a potential role of SelR in AD prevention.

## Methods

### Materials and Reagents

Matchmaker™ Gold yeast two-hybrid system, yeast strains Y2HGold and AH109, plasmids pACT2 and NpGBKT7, and human fetal brain cDNA library (using pACT2 as the vector) were purchased from Clontech Laboratories (Mountainview, CA, USA). Primary antibodies were from Santa Cruz Biotechnology (Santa Cruz, CA, USA). Secondary antibodies were purchased from Invitrogen (Carlsbad, CA, USA). N2aSW cells [17] were kindly provided by Professor Huaxi Xu and Yunwu Zhang in the Xiamen University. Dabsylated L-Met-SO [18] was kindly provided by Professor Herbert Weissbach of Florida Atlantic University.

### Gene Clone and Mutation

The *SelR* gene was cloned from the human fetus brain cDNA library. *SelR′* and *SelR′′* mutants were generated by site-directed mutation of the Sec residue in SelR to Cys and Ser, respectively [19]. Primers used and plasmids constructed in this paper are all presented in Table S1. All of the plasmids were confirmed to contain the target gene fragments by restriction enzyme analysis and DNA sequencing.

### Library Screening by Yeast Two-hybridization

SelR′ was used to screen the human fetal brain cDNA library via the yeast two-hybrid system. Yeast transformation and library screening were performed following the procedures described in the user manuals (Yeastmaker™ Yeast Transformation System 2 and Matchmaker™ Gold Yeast Two-hybrid System, Clontech). The screened positive prey plasmid was cotransformed with the bait plasmid into yeast for re-transformation verification [20] using the assay described in Method S1.

### FRET Analyses

HEK293T cells were co-transfected with pEYFP-C1-*Clu* and pECFP-C1-*SelR′* (or pECFP-C1-*Aβ_42_*) for FRET analysis using laser confocal microscope (Olympus FV1000, Tokyo, Japan). Two types of FRET methods, sensitized emission and receptor bleaching, which described previously[20]–[22], were adopted to measure FRET efficiency and the distance between the interactive proteins, and the details are described in Method S3.

### Co-IP Detection for Exogenous Protein Interaction

HEK293T cells were co-transfected with plasmids of HA-tagged *Clu* (or its fragments: *Clu_290–314_*, *Clu_315–381,_ Clu_382–460_*) and Myc-tagged *SelR′* (or *SelR′′*, *SelR_1–94_*, *SelR_19–82_*) or CFP-tagged *Aβ_42_*. Cell lysates were prepared as described previously [23]. Co-IP was performed using mouse anti-Myc or HA monoclonal antibody to bind Myc-tagged SelR′ or HA-tagged Clu, respectively, followed by protein A and G plus-agarose beads (Santa Cruz Biotechnology). The protein interacting with HA- or CFP-tag was detected by Western blot (WB) analysis using appropriate antibodies.

### Co-IP Detection for Endogenous Protein Interaction

Mouse cerebral cortex was isolated and washed triple with ice-cold PBS. Tissues were homogenized in RIPA lysis buffer, with 1 mM PMSF and sonicated on ice. Subsequently, the lysates were centrifuged at 12,000×g for 30 min at 4°C for immunoprecipitation. A proper amount of SelR antibody was added to the lysate (400 µg) and rotated overnight at 4°C, while the remaining protein was used as input. Protein A and G plus-agarose beads were added, and the mixture was rotated for another 3 h at 4°C.The samples were washed with RIPA buffer three times. Finally the beads were resuspended in sodium dodecyl sulfate (SDS) loading buffer and boiled for 5 min. After a short centrifugation step, the supernatant was collected for WB detection using the Clu antibody and proper second antibody.

### Pull-down Assay

For the GST pull-down assay, a total of 40 µl glutathione Sepharose FF beads (GE Healthcare, Waukesha, WI, USA) were incubated with 50 µg GST-fused Clu_315–381_, which was expressed in *E. coli* and purified by GST affinity chromatography for 1 h on ice in lysis buffer. The beads were then incubated with 50 µg His-tagged SelR′, which was expressed in *E. coli* and purified by His affinity chromatography for 2 h at 4°C. The beads were washed four times with lysis buffer, boiled in SDS loading buffer, and analyzed by WB using anti-His monoclonal antibody.

### Determination of Msrs Activity

Msrs activity was assayed using dabsylated L-Met-SO as a substrate. The assay for reducing dabsyl-Met-SO to dabsyl-Met was performed as described previously [24], and modifications are described in Method S4.

### Immunofluorescence Assay

Indirect immunofluorescence assay was performed to detect Clu protein in cells transfected with Myc empty vector or Myc-tagged *SelR′*, and the details are described in Method S5.

### Detection of Oxidative Stress

Intracellular ROS levels were determined using an ROS assay kit according to the manufacturer′s protocol. Cells transfected with target gene fragments were harvested and incubated with 10 µmol/L DCFH-DA (2′,7′-dichlorfluorescein-diacetate) at room temperature for 30 min in the dark and then analyzed using a flow cytometer (Beckman Coulter Altra, Brea, CA, USA).

### Statistical Analysis

Statistical analysis was performed using two-tailed Student′s t-tests, differences of *p*<0.01 and *p*<0.001 were considered significant and very significant, respectively. Data were expressed as the mean±SD of triplicate samples. All results were confirmed in at least three independent experiments.

## Results and Discussion

### Screening the Interacting Protein of SelR from the Human Fetal-brain cDNA Library

**Figure 1:**
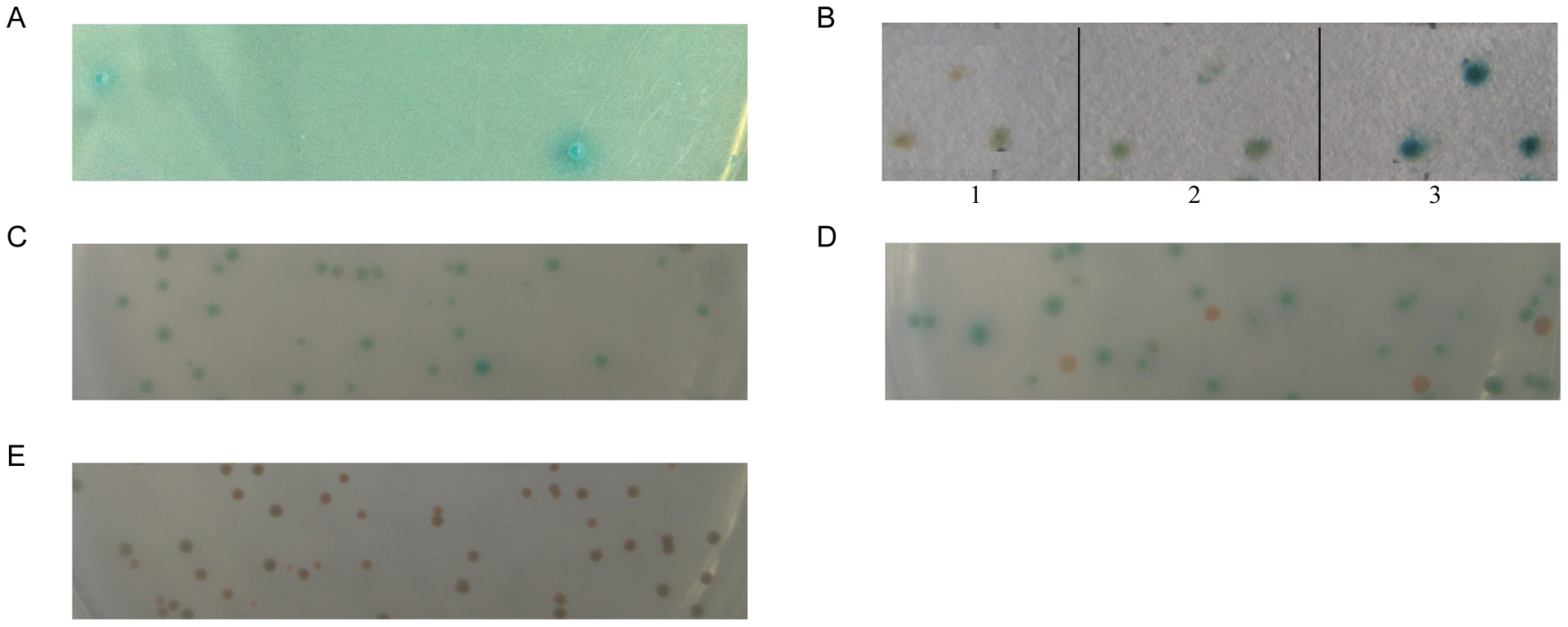
Using*SelR′* to screening the human fetal brain cDNA library with the yeast two-hybrid system. Plasmids carrying on the fetal brain cDNA library were co-transformed into the NpGBKT7-*SelR′*-containing yeast and screened by the selection plate for the blue colonies (A). The interaction between SelR′ and Clu was verified by re-transformation of the plasmids NpGBKT7-*SelR′* and pACT2-*Clu* into either AH109 (B) or Y2HGold (C) yeast cells. Yeast cells in B (1–3) were transformed with single NpGBKT7-*SelR′*, NpGBKT7-*SelR′* plus pACT2, and NpGBKT7-*SelR′* plus pACT2-*Clu* plasmids, respectively. Yeast cells in C, D, E were transformed with NpGBKT7-*SelR′* plus pACT2-*Clu*, pGBKT7-*p53* plus pADT7-*T* (positive control), and pGBKT7-*Lam* plus pADT7-*T* (negative control), respectively, followed by the selection on SD/−Leu/−Trp/X-α-Gal/Aba plates.

In this paper, human *SelR* gene was first mutated to *SelR′* by changing the Sec residue to a Cys. To determine whether *SelR′* was suitable to be a bait in Y2H system, tests for its toxicity and autoactivation in yeast cells were carried out. *SelR′* was used to construct the bait plasmid NpGBKT7-*SelR′* (BD plasmid) and transformed into Y2HGold yeast cells. No significant difference was observed in yeast growth between the *SelR′*-transformed and the negative control (pACT2-transformed) (data not shown), indicating that *SelR′* protein had no toxicity to the yeast cells. Meanwhile, yeast cells transformed with plasmid NpGBKT7-*SelR′*, grew in white color as the negative control (yeast cells co-transformed with pADT7-T and pGBKT7-Lam) (data not shown). NpGBKT7-*SelR′*-containing yeast was then used to screen the fetal brain cDNA library. Sixteen colonies were grown on five selection plates of quadruple dropout (SD/−Ade/−His/−Leu/−Trp) containing X-α-Gal and aureobasidin A (Aba)(Fig. 1A, a representative of the five selection plates). All plasmids extracted from the yeasts of those colonies were separately transformed into the *E. coli* Top10 cells to screen for colonies carrying the gene of the interactive protein (AD plasmid).

The BD plasmid and the screened AD plasmid were then re-transformed into two types of yeast cells, AH109 and Y2HGold. Deep blue spots were observed on filter paper blotted from the co-transformed AH109 cells using X-Gal as the chromagenic substrate of β-galactosidase (*LacZ* expression product) (Fig. 1B). For the co-transformed Y2HGold cells that use X-α-Gal as the substrate of α-galactosidase (*MEL1* expression product), large blue colonies were grown on SD/−Leu/−Trp/X-α-Gal/Aba selection plates (Fig. 1C). The AD plasmids in six positive colonies following re-transformation were sent out for DNA sequencing and bioinformatics analysis using NCBI’s non-redundant (nr) protein database. Three of them were identified to be Clu isoform 3 (ref|NP_001164609.1 or GENE ID: 1191).

Clu (as well known as apolipoprotein J) has multiple functions; it participates in cell apoptosis, cell cycle regulation, DNA repair, lipid transport, and cell adhesion [25], [26]. It is also linked to disease pathologies, including cancer and AD [27], [28]. Thus, we sought to verify the interaction between SelR and Clu through fluorescence resonance energy transfer (FRET), co-immunoprecipitation (co-IP), and pull-down assays.

### FRET Verification of the Protein Interaction

**Figure 2:**
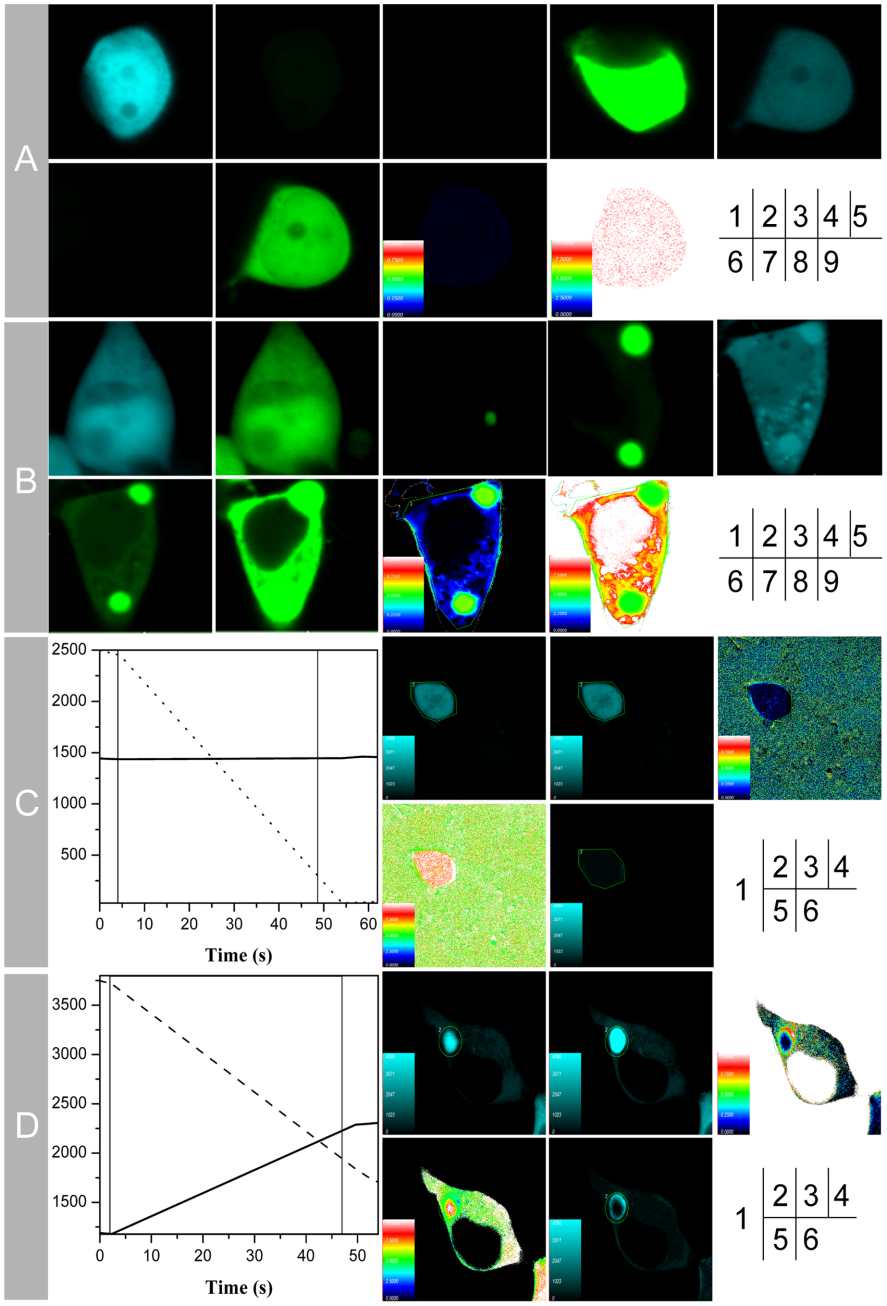
SelR′-Clu interaction verified by FRET techniques. (A&B) Protein interaction verified by the sensitized emission method of FRET. HEK293T cells were transfected with pECFP-C1, pEYFP-C1, pECFP-C1 plus pEYFP-C1 as negative controls (A), or transfected with pECFP-C1-*SelR′*, pEYFP-C1-*Clu*, pECFP-C1-*SelR′* plus pEYFP-C1-*Clu* for sample tests (B). Cells transfected with CFP/CFP- *SelR′* plasmids were excited at 405 nm and imaged in the CFP channel (1)/YFP channel (2). Cells transfected with YFP/YFP-*Clu* plasmids were excited at 405 nm (3)/515 nm (4) and imaged in the YFP channel. Cells co-transfected with CFP and YFP plasmids were excited at 405 nm and imaged in the CFP channel (5)/excited at 405 nm and imaged in the YFP channel (6)/excited at 515 nm and imaged at YFP channel (7), followed by FRET efficiency diagram (8) and the distance between donor and receptor (9). (C&D) Protein interaction verified by the receptor photobleaching method of FRET. HEK293T cells were co-transfected with the empty plasmids pECFP-C1 and pEYFP-C1 as a negative control (C) or co-transfected with pECFP-C1-*SelR′* and pEYFP-C1-*Clu* for sample tests (D). (1) Photobleaching curves (solid lines for donor fluorescence and dashed lines for receptor fluorescence). The region of interest (ROI) was bleached at 515 nm for 60 s. (2) The fluorescence images of donors (CFP/SelR′-CFP/) before bleaching. (3) The fluorescence images of donors after bleaching. (4) Donor fluorescence increments before and after bleaching. (5) Diagram of the distance between donor and receptor. (6) FRET efficiency diagram.

In order to determine whether the two proteins are interacting, two methods of FRET including sensitized emission and receptor photobleaching were performed. For the sensitized emission assay, the results were shown in Fig. 2 A &2B, and the images of them were acquired according to the information listed in Table S2. The energy transfer efficiency between CFP-SelR′ (donor) and YFP-Clu (receptor) shown in Fig. 2B(8) was calculated to be 40.0±9.2% (n = 10), and the distance between donor and receptor shown in Fig. 2B(9) was calculated to be 5.7±0.4 nm (n = 10). FRET was undetectable for control cells co-transfected with empty vectors pECFP-C1 and pEYFP-C1 (Fig. 2A(6)). FRET efficiency was shown in Fig. 2A(8) and calculated to be 1.2±1.0% (n = 10) with a distance of 9.8±0.1 nm (n = 10) shown in Fig. 2A(9).

Results from the acceptor photobleach experiments showed that fluorescence of the CFP-SelR′ donor was significantly increased after the receptor was bleached (Fig. 2D(4)), but this was not observed in control cells (Fig. 2C(4)). The distance between CFP-SelR′ donor and YFP-Clu receptor is shown in Fig. 2D(5) and was calculated to be 5.8±0.5 nm (n = 18). The energy transfer efficiency between CFP-SelR′ and YFP-Clu is shown in Fig. 2D(6) and was calculated to be 41.8±7.6% (n = 18). As for control cells co-transfected with empty vectors pECFP-C1 and pEYFP-C1, FRET efficiency was calculated to be 2.9±1.8% (n = 3) (Fig. 2C(6)), and the distance was 9.5±0.3 nm (n = 3, Fig. 2C(5)). All FRET results confirmed the interaction between SelR′ and Clu.

### Co-IP Verification of the Protein Interaction

**Figure 3:**
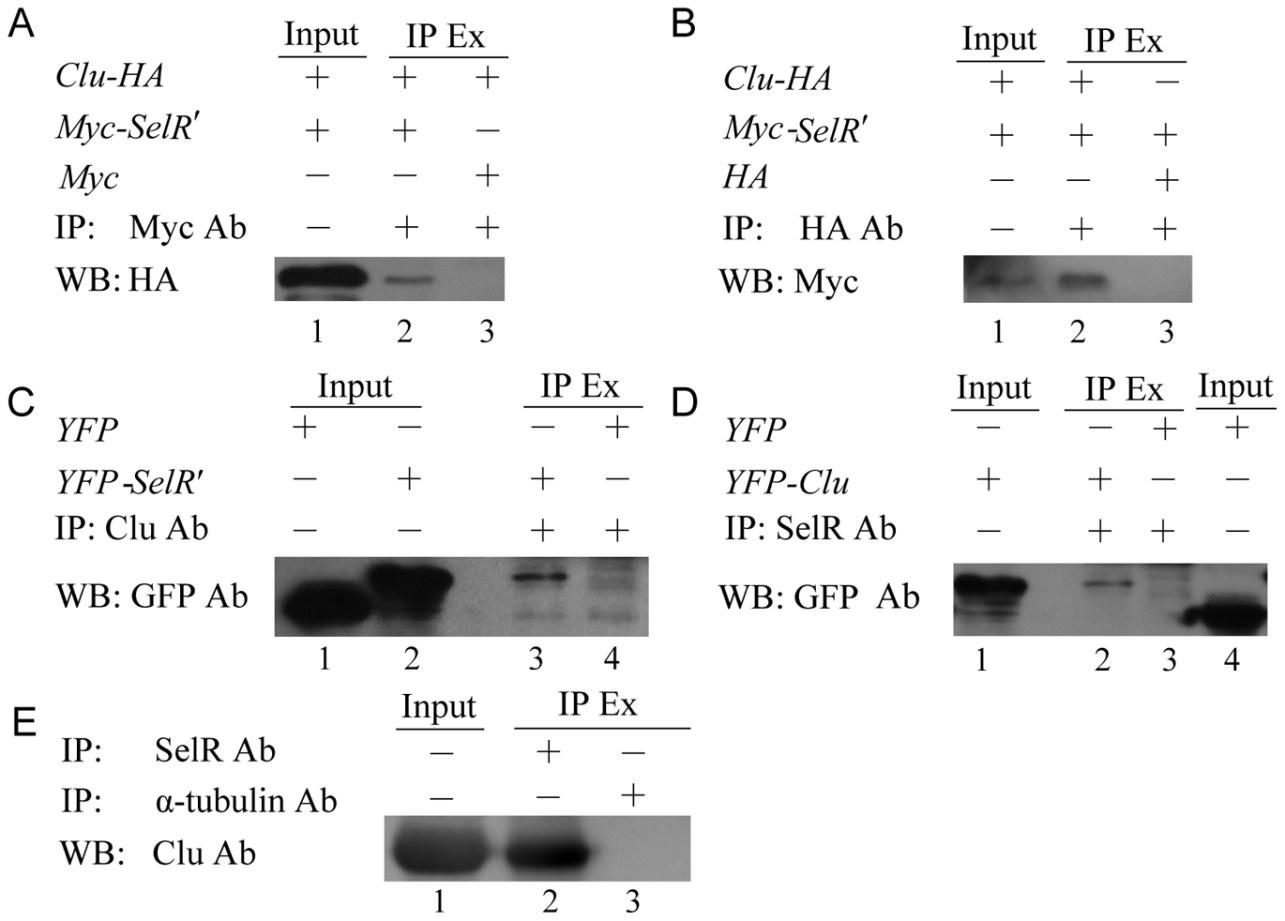
SelR-Clu interaction verified by gene-transfected and endogenous proteins co-IP assays. (A&B) Two-genes co-transfected co-IP. HEK293T cells were co-transfected with pCMV-Myc-*SelR*′ and pcDNA3.1-HA-*Clu* plasmids. Cell lysates were analyzed by immunoprecipitation (IP) and Western blot (WB) using Myc-tag (A) and HA-tag (B) antibodies. 5% cell lysates (input) was added in the gel in each experiment to be a positive control. HEK293T cells co-transfected with plasmids pCMV-Myc and pcDNA3.1-HA-Clu or pcDNA3.1-HA and pCMV-Myc-SelR*′* were used as the negative controls for (A) and (B) respectively. (C&D) Single-gene transfected co-IP. HEK293T cells were transfected with pECFP-C1-*SelR′* plasmids (C) or pEYFP-C1-*Clu_290–460_* (D). Cell lysates were analyzed by IP with Clu antibodies (C) or SelR antibody (D), followed by WB with GFP antibody. Cells transfected with pEYFP-C1 plasmids were used as the negative control for both (C) and (D). (E) Endogenous proteins co-IP. Mousee cerebral lyastes were immunoprecipitated with SelR antibody followed by WB with Clu antibody. Endogenous α-tublin was used as a negative control. Ab: Antibody; IP Ex: IP extract.

Co-IP was performed to further study the interaction between SelR′ and Clu in mammalian cells. For exogenous co-IP, pcDNA3.1-*Clu_315–381_-HA* and pCMV-Myc-*SelR′* plasmids were co-transfected into HEK293T cells. An antibody against Myc was used to IP Myc-tagged SelR′ from cell extracts. The isolated proteins were analyzed by WB using an anti-HA antibody. A specific association between Myc-tagged SelR′ and HA-tagged Clu is shown in lane 2 of Fig. 3A. Conversely, a HA antibody was used to IP HA-tagged Clu, and Myc antibody was used to probe for Myc-tagged SelR′. The association of Clu with SelR′ was also detected in lane 2 of Fig. 3B.

In order to investigate if the protein interaction was endogenous, we performed single-gene trasfected endogenous co-IP and non-gene transfected endogenous co-IP assays. For single-gene transfected endogenous co-IP, pEYFP-C1-*SelR′*, pEYFP-C1, and pEYFP-C1-*Clu* plasmids were transfected into HEK293T cells, and an antibody against Clu or SelR was used to IP endogenous Clu in pEYFP-C1-*SelR′* transfected cells or endogenous SelR in pEYFP-C1-*Clu* transfected cells. Western blotting was performed using an antibody against GFP (the same antibody against CFP or YFP), and the results showed specific association of YFP-tagged SelR′ with endogenous Clu (Fig. 3C, lane 3) and YFP-tagged Clu with endogenous SelR (Fig. 3D, lane 2) using pEYFP-C1 transfected cells as a negative control. For non-gene transfected endogenous co-IP, SelR was immunoprecipitated from the mouse cerebral cortex lysates using a SelR antibody, and the Clu in the precipitates were detected by WB. The group using an α-tublin antibody to immunoprecipitate endogenous α-tublin from the lysates, and then detecting the Clu in the precipitates by WB was used for the negative control. The results showed that the interaction between SelR and Clu could happen at endogenous protein levels (Fig. 3E, lane 2).

### Identification of the Interacting Domains

**Figure 4:**
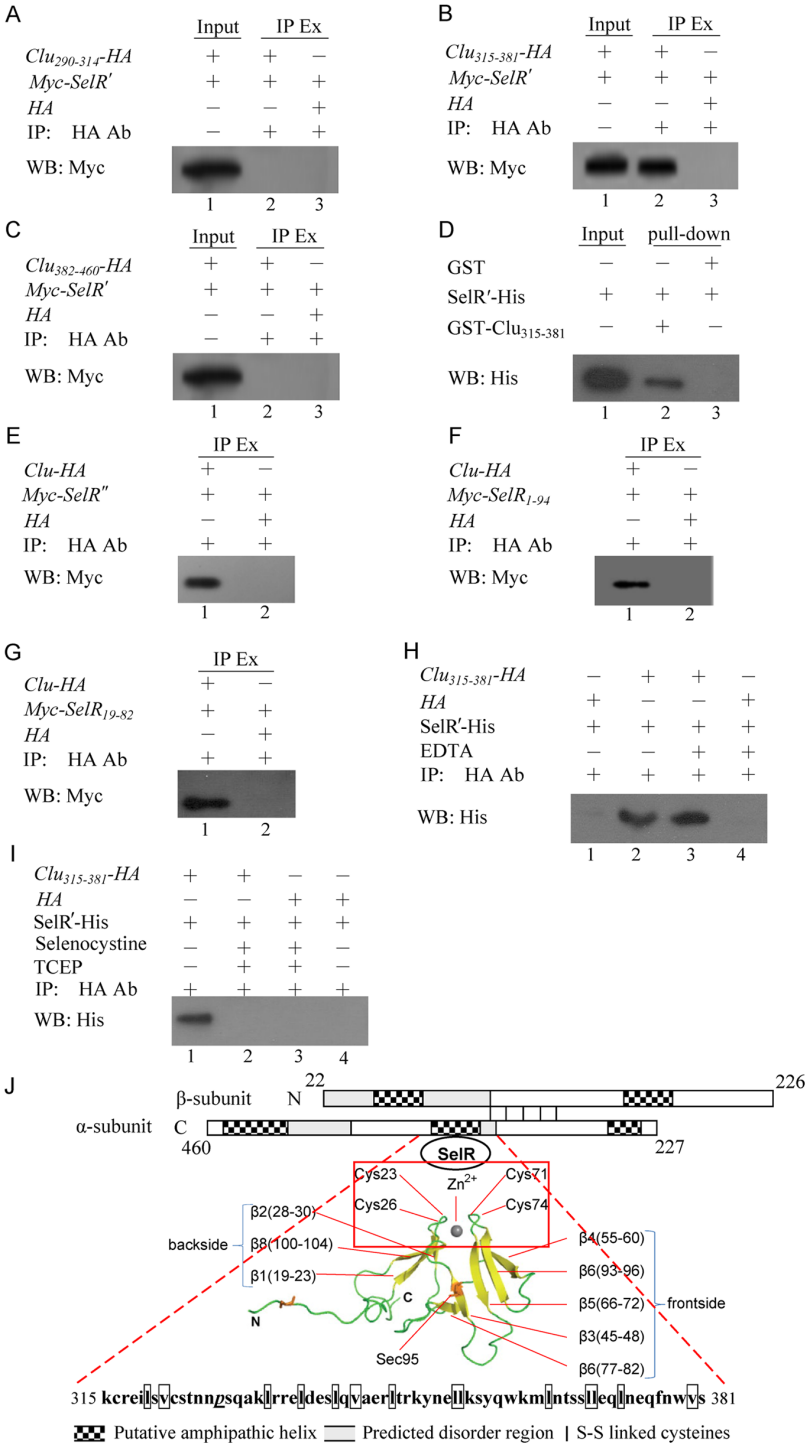
Identification of SelR and Clu interacting domains. (A–C) Co-IP analyses of the interacting domains of Clu which interacted with SelR. HEK293T cells were co-transfected with different plasmid pairs (pCMV-Myc-*SelR′* and pcDNA3.1-*Clu_290–314_-HA*(A), pCMV-Myc-*SelR′* and pcDNA3.1-*Clu_315–381_-HA* (B), pCMV-Myc-*SelR′* and pcDNA3.1-*Clu_382–460_-HA* (C)). Cell lysates were analyzed by IP with HA-tag antibody and WB with Myc-tag antibody. 5% cell lysate (input) was added in a gel to be a positive control. (D) Pull-down analysis of the interaction between GST-*Clu_315–381_* and SelR. GST-Clu_315–381_ was immobilized on glutathione Sepharose FF beads and its binding protein His-tagged SelR′ was analyzed by WB with His-tag antibody. GST was used as a negative control. (E–G) Co-IP analysis of SelR mutant and fragments interacting with Clu. HEK293T cells were co-transfected with plasmid pairs pcDNA3.1-HA-*Clu* and pCMV-Myc-*SelR′′*(E) or pCMV-Myc-SelR1-94 (F), or pCMV-Myc-SelR19-82 (G). Cell lysates were analyzed by IP with HA-tag antibody and WB with Myc-tagged antibody. (H&I) Pull-down analyses on the function of zinc ion in maintaining the interaction between SelR′-His and Clu_315–381_-HA. Clu_315–381_-HA was immobilized on the beads containing HA-tagged antibody. SelR′-His was then added to bind with Clu_315–381_. Metal chelating agents EDTA (H) and Selenocystine (I) were tested to remove the zinc ion bound to the tetrahedral of SelR′. (J) Model of the Clu and SelR interaction. The two Clu subunits are linked by five disulfide bonds. The shaded and hatched squares represent predicted disordered regions and predicted amphipathic helices, respectively. The central region of Clu_315–381_ was shown to interact with the tetrahedral structure consisting of a zinc ion binding with four cysteine residues [Zn^2+^(Cys)_4_] in SelR. Ab: Antibody; IP Ex: IP extract.

Clu is comprised of two subunits, α- and β-, which contain three long regions of natively disordered domains combined with amphipathic α-helical structures (Fig. 4J) [29]. One of those regions forms a dynamic, molten globule-like binding site that provides Clu the ability to bind to a variety of molecules [30]. Using the yeast two-hybrid system, a 171-amino-acid (aa)-length Clu fragment located between residues 290 and 460 was screened out to interact with SelR′. To further map the region of Clu that directly binds SelR′, the online program PredictProtein [31] was used to analyze Clu′s secondary protein structure. The bioinformatics results were compared with previous reports [32], [33] to predict the potential SelR binding domain. The available Clu fragment was then separated into three regions, spanning aa 290–314, aa 315–381, and aa 382–460. Those regions were amplified via polymerase chain reaction and inserted into the pcDNA3.1-HA vector to construct pcDNA3.1-*Clu_290–314_-HA*, pcDNA3.1-*Clu_315–381_-HA*, and pcDNA3.1-*Clu_382–460_-HA* plasmids. Co-IP was performed by co-transfecting HEK293T cells with Myc-*SelR′* and each Clu fragment. As shown in Fig. 4A–C, only the middle part of Clu_315–381_ was found to interact with SelR′ (Fig. 4B, lane 2).

To corroborate the interacting domain of Clu, we performed a glutathione-S-transferase (GST)-pull-down on the recombinant peptide expressed from the middle part of Clu_315–381_ in combination with His-tagged SelR′. Then the isolated proteins were analyzed by Western blotting using the antibody against His. Similar to the co-IP results, Clu_315–381_ was found to strongly bind SelR′ (Fig. 4D, lane 2). These results indicate that the central region of Clu spanning aa 315–381 is required for binding SelR′; it also indicates that the interaction between them is direct and that no other proteins are involved.

Interestingly, the SelR binding site of Clu corresponds to the molten globule domain in the middle of the α-subunit, while the other two globule regions are situated in the N- and C-terminals. Sequence analysis revealed that Clu_315–381_ is rich in valine (V) and leucine (L) commonly found in coiled-coil helices, but proline (P) residues and glycine (G), which are the two strongest helix breaking residues, are rare and absent, respectively. This region has already been reported to interact directly with Chibby and prion proteins [32], [33].

In order to investigate the interacting domain, SelR was mutated to SelR′. As the change of Sec to Cys generally does not alter selenoprotein structure, the biochemical properties of SelR′ are similar to SelR. However, this is not true when Sec was mutated to a serine residue (Ser) to produce the pCMV-Myc-*SelR*′′ plasmid. Co-IP was performed by co-transfecting cells with *SelR*′′ and *Clu* (Fig. 4E). Interestingly, SelR′′ also interacted with Clu (lane 1 in Fig. 4E). We also constructed a Myc-tagged SelR truncate (*SelR_1–94_*) in which the intercepted SelR segment stopped at the Sec-coding TGA (Sec95). Co-IP was performed by co-transfecting cells with *SelR_1–94_* and *Clu* (Fig. 4F). We also detected interaction between SelR truncate and Clu (lane 1 in Fig. 4F).

The mobile N-terminus region of SelR is important to the protein’s structure, and the resolving Cys4 is associated with Se-based enzymatic catalysis. To investigate if the catalytic center and the protein binding domain of SelR are different, co-IP was performed by co-transfecting cells with *Clu* and *SelR_19–82_*, a fragment of *SelR* lacking of the catalytic center of Sec95 and the flexible N-terminus (Fig. 4G). Results showed that SelR_19–82_ could also interact with Clu (lane 1 in Fig. 4G). As SelR′, SelR′′, SelR_1–94_, and SelR_19–82_ all interacted with Clu, it is clear that the interaction between SelR and Clu is not dependent on the Sec residue, the C-terminal (aa 95–116), or the N-terminal (aa 1–18) of SelR.

It has been reported that the overall SelR structure consists of two anti-parallel β-sheets [34]. The first sheet has three strands that form the back of the structure, while the second has five strands forming the front and contains the active site of SelR (Sec95). The back β-sheet is constructed by strand β1 (aa 19–23), β8 (aa 100–104), and β2 (aa 28–30). The flexible C-terminal region comes out of the middle of the back β-sheet. The front β-sheet is connected in the following order: β3 (aa 45–48), β7 (aa 93–96), β6 (aa 77–82), β5 (aa 66–72), and β4 (aa 55–60) and forms the protein’s hydrophobic core through residues Leu67, Val69, Phe94, and Ile96 linking to the back β-sheet hydrophobic amino acids Tyr21, Phe31, and Phe103 at the bottom of the structure. The top portion of SelR is held together through tetrahedral structural zinc, in which the metal ion is bound coordinately to the protein matrix by Cys_23_, Cys_26_, Cys_71_, and Cys_74_.

In our experiments, mutating Sec95 to Cys95/Ser95, truncation at Sec95, and deletion of both N−/C- terminals did not affect the interaction between SelR and Clu. Because both β-sheets were significantly disturbed by the changes above, and the only undisturbed part was the tetrahedral [Zn(Cys)_4_]^2+^ structure located in the top portion of SelR, it is reasonable to deduce that it contains the potential domain for SelR to interact with Clu. To test this hypothesis, we employed ligands to remove the Zn^2+^ ion in SelR to destroy the tetrahedron. We found that ethylenediaminetetraacetic acid (EDTA), which is a weaker Zn^2+^-binding ligand than Cys, could not destroy the tetrahedral structure (Fig. 4H) or disturb the interaction between SelR′ and Clu (lane 3 in Fig. 4H). However, Sec, which is a stronger Zn^2+^-binding ligand than Cys, destroyed both the tetrahedral structure and the protein interaction (lane 3 in Fig. 4I). Thus, we propose that SelR uses its zinc-bound structure to interact with Clu, leaving the catalytic Sec95 residue and resolving Cys4 residue to carry out the enzyme’s catalytic functions. Fig. 4J shows the model of Clu interacting with SelR′.

### Mutual Effect between the Interactive Proteins

**Figure 5:**
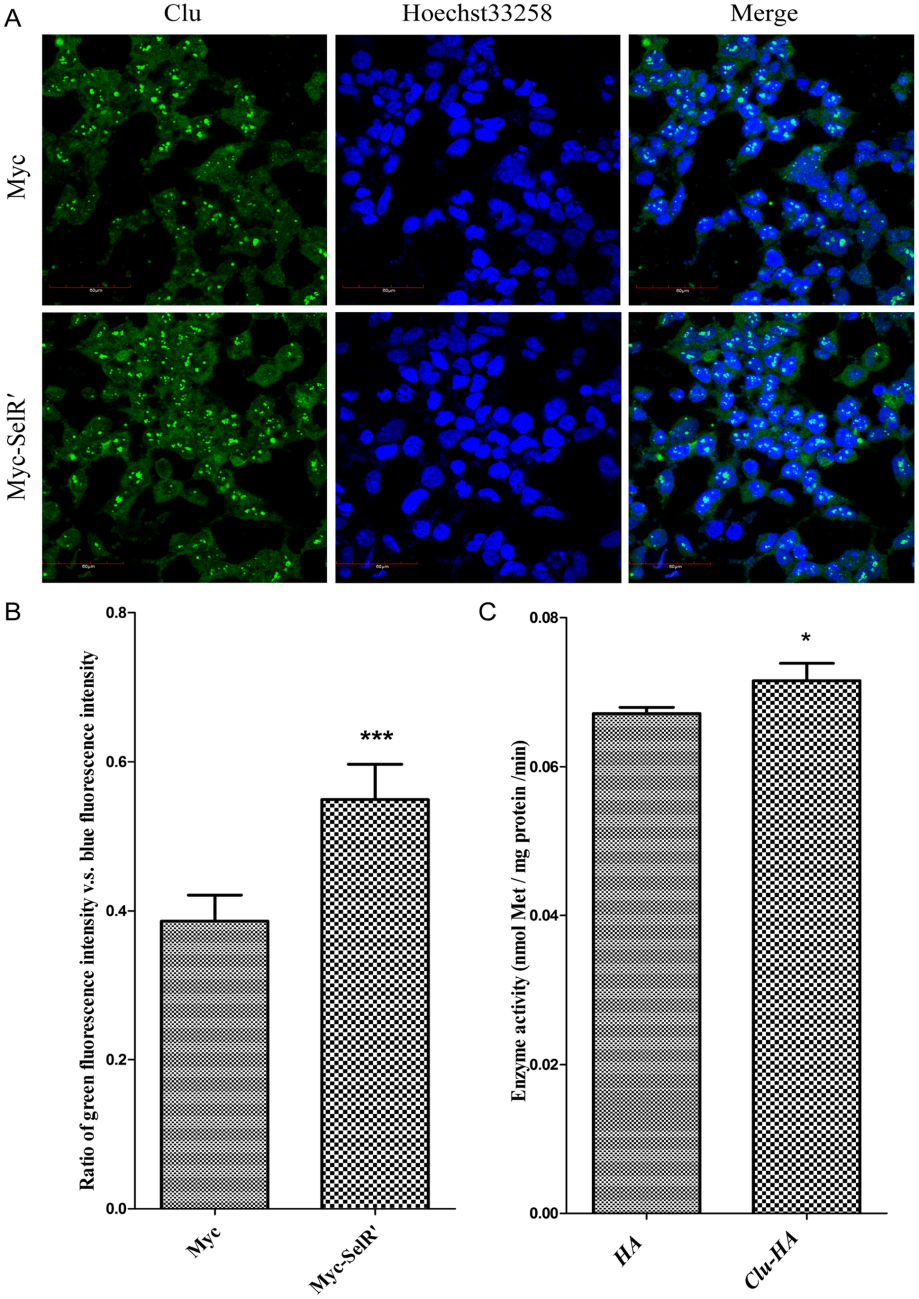
Effects of SelR and Clu overexpression on Clu levels and SelR activity. (A&B) Effect of SelR overexpression on Clu levels. HEK293T cells were transfected with pCMV-Myc or pCMV-Myc-SelR′ plasmids. Immunofluorescence microscopy of Clu (green) was observed together with cell nuclei stained by Hoechst 33258 (blue) (A). Fluorescence intensity was calculated using Olympus Fluoview FV1000 Toolbox software. Ratio of green *vs*. blue fluorescence intensity was shown in (B) (*** *P*<0.001). (C)Effect of Clu overexpression on SelR activity, HEK293T cells were transfected with pcDNA3.1-HA or pcDNA3.1-HA-*Clu_290–449_* plasmids. Cell lysates were used for the detection of methionine sulfoxide reductase activity of SelR by HPLC (*P<0.05).

Clu is an enigmatic molecule associated with various physiological processes and diseases. Its protein levels can be affected by different modes of cellular stress, numerous growth and cytokines, and some oncogenes [35], [36]. In order to find out the effect of SelR overexpression on Clu protein level, HEK293T cells were transiently transfected with SelR′ and immunofluorescently assessed for Clu expression using an Clu antibody. As shown in Fig. 5A and B, transfection of SelR increased intracellular Clu. A separate group of HEK293T cells were transiently transfected with *Clu-HA*, and SelR activity was assessed. Fig. 5C demonstrates that intracellular SelR activity was increased due to Clu overexpression. According to previous reports, Clu can prevent Aβ aggregation by blocking the synthesis of Aβ_42_ peptides or increasing Aβ solubility, while SelR can reduce Met sulfoxide to Met and reduce oxidative stress. Therefore, it is reasonable to infer that SelR can interfere with AD pathogenesis by increasing Clu expression to prevent Aβ aggregation, and this in turn promotes SelR activity to block oxidative stress that facilitate the onset and development of AD. These results suggest that the interaction between SelR and Clu could play an important role in AD prevention.

### In vitro and in vivo Effects of SelR-Clu Interaction on SelR Activity

**Figure 6:**
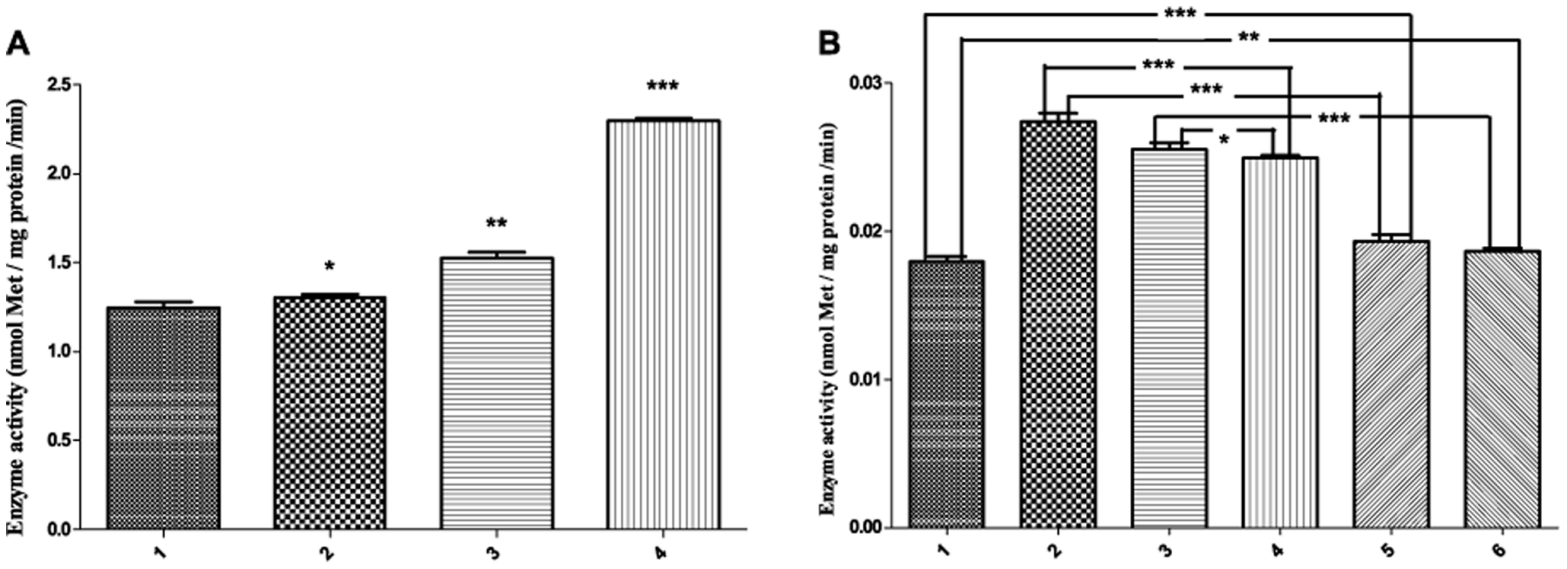
Effect of the SelR-Clu interaction on SelR activity. (A) Effect of SelR-Clu interaction on SelR activity in vitro. The mole ratios of Clu against SelR′ in columns 1–4 were 0, 0.4, 0.8, and 2, respectively. The methionine sulfoxide reductase activity of SelR was measured by HPLC. (B) Effect of SelR-Clu interaction on SelR activity in vivo. Columns 1–6 represented N2a cells co-transfected with plasmid pairs of pcDNA3.1-HA and pCMV-Myc (HA+Myc), pCMV-Myc-*SelR′* and pcDNA3.1-HA-*Clu*_290–449_ (SelR′+Clu_290–449_), pCMV-Myc-*SelR′* and pcDNA3.1-*Clu_315–381_-HA* (SelR′+Clu_315–381_), pCMV-Myc-*SelR′* and HA empty vector (SelR′+HA), Myc empty vector and pcDNA3.1-*Clu_290–449_-HA* (Myc+Clu_290–449_), Myc empty vector and pcDNA3.1-*Clu_315–381_-HA* (Myc+ Clu_315–381_), respectively.

SelR is a member of the MsrB family that specifically catalyzes the reduction of R-form Met sulfoxide in proteins and participates in repairing oxidatively damaged proteins. To gain insight into the influence of the interaction between SelR and Clu on SelR activity, in vitro and in vivo experiments were performed. The in vitro results revealed that increasing the quantity of Clu in the reaction mixture dose-dependently promoted the enzyme activity of SelR′ (Fig. 6A). In vivo experiments were performed in mouse neuroblastoma (N2a) cells co-transfected with *SelR′* and different *Clu* fragments, using different combinations of empty plasmids as controls (e.g., empty vectors HA+Myc, *SelR′*+*Clu_290–449_*, *SelR′*+*Clu_315–381_*, *SelR′*+HA, Myc+C*lu_290–449_*, Myc+*Clu_315–381_*). Intracellular SelR activity was measured using protein extracts from the above groups of cells (Fig. 6B). Among them, cells co-transfected with *SelR′* and *Clu_290–449_* gene fragments had the highest SelR activity, which was significantly higher than the two control groups (cells co-transfected with HA+Myc or Myc+*Clu_290–449_*). The second highest SelR activity was measured in cells co-transfected with *SelR′* and *Clu_315–381_*, this was also significantly higher than the two control groups (cells co-transfected with HA+My*c* or Myc+*Clu_315–381_*). Cells co-transfected with *SelR′* gene and HA empty vector had the third highest enzyme activity, which supports the conclusion from in vitro experiments that Clu could increase SelR activity. Compared with HA+Myc-co-transfected cells, those co-transfected with Myc+*Clu_290–449_* or Myc+*Clu_315–381_* had higher endogenous SelR activity due to Clu fragment expression. Collectively, both the in vitro and in vivo results support the hypothesis that Clu promotes SelR activity.

### SelR-Clu Interaction Decreases Intracellular ROS

**Figure 7:**
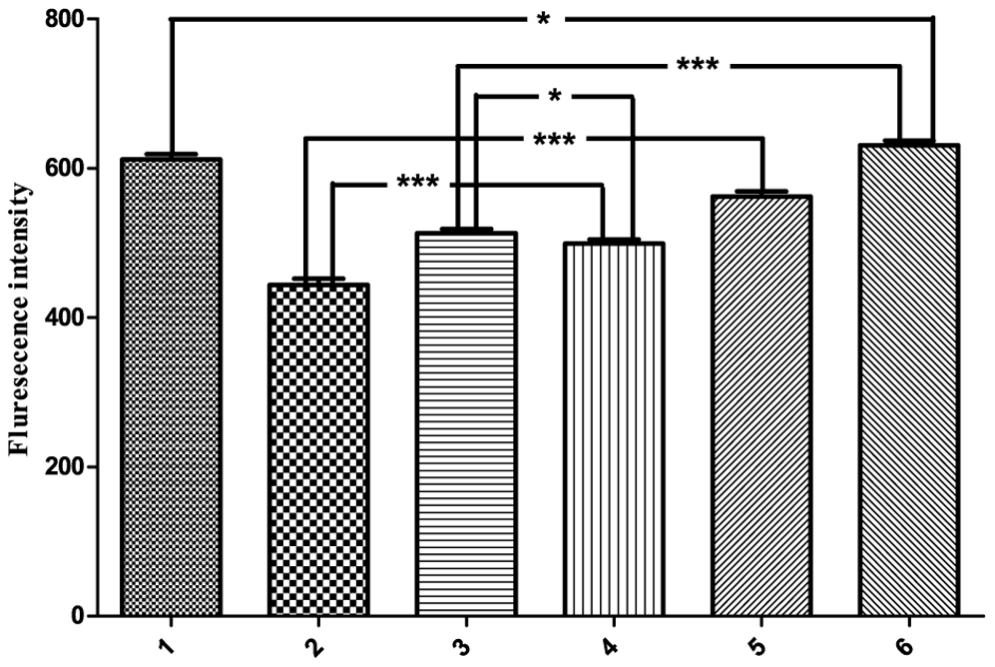
Effect of SelR- Clu interaction on intracellular ROS levels. N2aSW cells co-transfected with different plasmid pairs and incubated 36 h before they were harvested for ROS detection using DCFH-DA assay. Columns 1–6 represented N2aSW cells co-transfected with the plasmid pairs shown in Fig. 6(B).

SelR specifically catalyzes the reduction of R-form Met sulfoxide in proteins, thus decreasing intracellular ROS. Clu plays dual roles in regulating cell apoptosis; secreted Clu (sClu) prevents cell apoptosis, but nuclear Clu (nClu) promotes it. Research indicates that siRNA-mediated Clu gene silencing in cancer cells can significantly reduce cellular growth and increase rates of spontaneous endogenous apoptosis [37]. Studies have revealed that nClu acts via a putative BH3 motif (aa 319–379) in its C-terminal coiled coil (CC2) domain (aa 323–330) to sequester Bcl-XL, release Bax, and promote apoptosis [38]. N2aSW is a cell line stably expressing human amyloid precursor protein containing the Swedish mutation to promote extracellular Aβ accumulation [39]. This process generally increases intracellular ROS. Therefore, we measured intracellular ROS levels in N2aSW cells co-transfected with SelR and different Clu fragments (Fig. 7). Contrary to SelR activity, cells co-transfected with *SelR’* and *Clu_290–449_* had significantly lower ROS levels than both control groups (cells co-transfected with HA+Myc or Myc+*Clu_290–449_*). Similarly, cells co-transfected with SelR′ and *Clu_315–381_* also had lower ROS level than their two controls (co-transfected with HA+Myc or Myc+*Clu_315–381_*), but levels close to those co-transfected with *SelR′* and HA empty vector. This phenomenon can be explained by the structural feature of the Clu_315–381_ fragment, which contains the very important BH3 domain. Proteins containing a BH3 domain can be activated by different kinds of cell irritants and play a pivotal role in the process of mitochondrial apoptosis [40] and the subsequent increase of intracellular ROS. This is likely why ROS levels were significantly higher in cells co-transfected with *Clu_315–381_* and Myc empty vector. It also explains why intercellular ROS levels in cells co-transfected with *SelR′* and *Clu_315–381_* are similar to those co-transfected with *SelR′* and HA empty vector.

As shown in Fig. 6B, interaction between SelR and Clu can significantly increase intracellular SelR activity in N2a cells. However, no significant increase of SelR activity was observed in N2aSW cells co-transfected with SelR′ and Clu (data not shown). This could be explained by a decrease in ROS levels mediated by SelR. Based on the results described above, we conclude that the interaction between SelR and Clu increases the Msr activity of SelR, which increases ROS scavenging and redox balance in vivo.

### Interaction between Clu and Aβ_42_ Verified by FRET and co-IP

**Figure 8:**
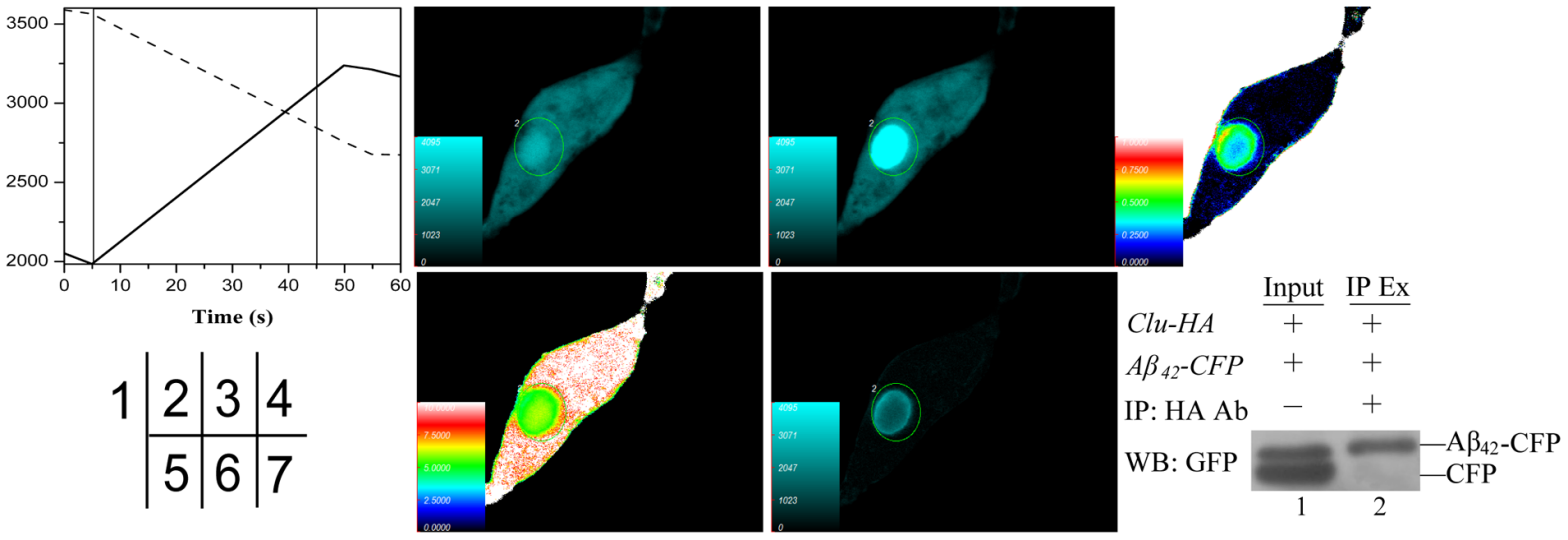
Interaction of Aβ_42_ with Clu verified by FRET and co-IP. (1–6) The receptor photobleaching method of FRET. HEK293T cells were co-transfected with pcDNA3.1-*Aβ_42_-CFP* and pEYFP-C1-*Clu* for sample tests, while those co-transfected with pECFP-C1 and pEYFP-C1 were used as the negative control (shown in Fig. 2(C)). (1) Photobleaching curves (solid lines for donor fluorescence and dashed lines for receptor fluorescence). (2) The fluorescence images of donors (Aβ_42_-CFP before bleaching. (3) The fluorescence images of donors after bleaching. (4) Donor fluorescence increment before and after bleaching. (5) Diagram of the distance between donor and receptor. (6) FRET efficiency diagram. (7) Co-IP analysis. HEK293T cells were co-transfected with pcDNA3.1-*Aβ_42_-CFP* and pcDNA3.1-*Clu-HA* plasmids. The cell lysates were then analyzed by IP with HA-tag antibody and WB with GFP antibody. 5% cell lysates (input) was used as a positive control. Ab: Antibody; IP Ex: IP extract.

Clu reportedly co-localizes with fibrillar deposits in systemic and cerebral amyloid disorders [41]. It demonstrates high affinity with Aβ to form stable 1∶1 stoichiometric complexes [42]. Therefore, we investigated the possibility for Clu to interact with Aβ using FRET and co-IP assays. Cells co-transfected with pcDNA3.1-*Aβ_42_-CFP* (constructed in the reference [43])and pEYFP-C1-*Clu* emitted cyan fluorescence in CFP and YFP channels, respectively, with 405 nm and 515 nm excitation. FRET was detected as shown in Fig. 8(1–6). After photobleaching YFP-*Clu*, increased *Aβ_42_*-CFP fluorescence was observed in the region of acceptor bleaching compared with pre-bleaching images (Fig. 8(2–3)). The energy transfer efficiency between Aβ_42_-CFP and YFP-Clu is shown in Fig. 8(6) and was calculated to be 33.7±7.5% (n = 17). The distance between the two proteins is shown in Fig. 8(5) and was calculated to be 6.3±0.5 nm (n = 17). A negative control was assessed by co-transfecting empty vectors pECFP-C1 and pEYFP-C1 into the cells for FRET detection (Fig. 2C). We also performed a co-IP assay to verify the interaction between *A*β_42_ and Clu in mammalian cells. pcDNA3.1-*Clu-HA* and pcDNA3.1-*Aβ_42_-CFP* plasmids were co-transfected into HEK293T cells. An antibody against HA was used to immunoprecipitate HA-tagged Clu from cell extracts. The isolated proteins were analyzed by WB using an antibody against CFP, and the result showed specific association of CFP-tagged *A*β_42_ with HA-tagged Clu (Fig. 8(7), lane 2).

Recently, large-scale population surveys have shown that Clu is linked with AD [15], [16]. Clu, an extracellular chaperone, is capable of preventing the precipitation of several proteins, including Aβ peptide under denaturing conditions, and it also slows down Aβ_1–42_ aggregate formation by cooperating with apolipoprotein E to function as a neuroprotective and anti-amyloidogenic molecule [28]. It can also transport the Aβ peptide into biological fluids, maintain its solubility, and modulate its movement across the blood-brain barrier [42]. Aβ neurotoxicity is reportedly caused by the oligomeric, rather than the fibrillar form. Chaperone molecules can reduce Aβ neurotoxicity by accelerating the aggregation of peptides in solution [44]. Clu may function in this way to protect neurons from Aβ oligomers.

SelR plays an important role in preventing oxidative damage to the brain, and this is an important factor in a variety of neurological diseases. The 35^th^ amino acid on the Aβ peptide is Met. Oxidation of Met^35^ in Aβ generates free radicals. Ultimately, free radical production in the brain may overwhelm the antioxidant defence system, and can promote Aβ aggregation and neurotoxicity. SelR can reduce oxidized Met^35^, which maintains the redox balance, prevents Aβ aggregation, and interferes with AD development. Our results indicate that the central region of Clu (aa 315–381) contains a dynamic molten domain with an amphipathic-helix that interacts with the tetrahedral structure of SelR, which consists of a Zn^2+^ ion binding with four cysteine residues to form a [Zn^2+^(Cys)_4_] complex. The direct interaction between SelR and Clu increases SelR activity and reduces intracellular ROS in N2aSW cells. Whether these results are closely related to the reduction of the oxidized Met^35^ of Aβ or whether this pathway is involved in Aβ aggregation requires further investigation. However, the findings in this paper provide new insights into the molecular mechanisms of SelR and its possible role in AD pathogenesis.

## Supporting Information

Table S1 Primers used and plasmids constructed. Underlined sequences are DNA restriction endonuclease digestion sites.
(DOCX)

Table S2 Information on the images acquired through the sensitized-emission method.
(DOCX)

Method S1 **Confirmation of positive interaction in yeast.**
(DOCX)

Method S2 **Protein expression and purification.**
(DOCX)

Method S3 **FRET analyses.**
(DOCX)

Method S4 **Determination of methionine sulfoxide reductase activity.**
(DOCX)

Method S5 **Immunofluorescence assay.**
(DOCX)
