# Supplementary material for: Direct Interaction of Selenoprotein R with Clusterin and Its Possible Role in Alzheimer’s Disease
Source: PLoS One. 2013 Jun 21;8(6):e66384. doi: 10.1371/journal.pone.0066384 (PMC3689823; doi:10.1371/journal.pone.0066384)
Supplement: Table S1 — Primers used and plasmids constructed. Underlined sequences are DNA restriction endonuclease digestion sites. (DOCX) [file pone.0066384.s001.docx]

| Primer | Sequence | Restriction site | Gene fragment | Plasmid constructed |
| --- | --- | --- | --- | --- |
| F1  R2 | 5′-***TT***GGCCATTACGGCCATGTCGTTCTGCAGCTTCTTCGGGG -3′  5′-GCTGAATATGCAGAATCGGG  A-3′ | *SfiI* | SelR ORF creating the mutation site from TGA to TGC | NpGBKT7-*SelR′*  pCMV-MYC-*SelR′* |
| F2  R1 | 5′-TCCCGATTCTGCATATTCAG  C-3′  5′-***TT***GGCCGAGGCGGCCCTAGTGACCCTGGGAGGCAGAAGTT-3′ | *SfiI* |  |  |
| F1  R3 | 5′-***TT***GGCCATTACGGCCATGTCGTTCTGCAGCTTCTTCGGGG -3′  5′-GCTGAATATGGAGAATCGGG  A-3′ | *SfiI* | SelR ORF creating the mutation site from TGA to TCC fused MYC | pCMV-MYC-*SelR′′* (Myc-tagged *SelR′′*) |
| F3  R1 | 5′-TCCCGATTCTCCATATTCAG  C-3′  5′-***TT***GGCCGAGGCGGCCCTAGTGACCCTGGGAGGCAGAAGTT-3′ | *SfiI* |  |  |
| F4 | 5′-***CG***GAATTCTATGGACGATGACCGGACTGTGTGCC -3′ | *EcoRI* | Clu ORF spanning amino acids 290-460 | pEYFP-C1-*Clu_290-460_* (YFP-tagged *Clu_290-460_*) |
| R4 | 5′-***CG***GGATCCTCACTCCTCCCGGTGCTTTTTGCGG -3′ | *BamHI* |  |  |
| F5 | 5′-***CG***GAATTCTATGTCGTTCTGCAGCTTCTTCGGGG -3′ | *EcoRI* | SelR ORF creating the mutation site from TGA to TGC fused CFP | pECFP-C1-*SelR′* (CFP-tagged *SelR′*) |
| R5 | 5′-***CG***GGATCCCTAGTGACCCTGGGAGGCAGAAGTT -3′ | *BamHI* |  |  |
| F6 | 5′-***TT***GGCCATTACGGCCATGGACGATGACCGGACTGTGTG -3′ | *SfiI* | Clu ORF spanning amino acids 290-460 | pcDNA3.1-HA-*Clu* |
| R6 | 5′-***TT***GGCCGCCTCGGCCTCACTCCTCCCGGTGCTTTTTGCGG -3′ | *SfiI* |  |  |
| F7 | 5′-***TT***GGCCATTACGGCCATGGACGATGACCGGACTGTGTGCC -3′ | *SfiI* | Clu ORF spanning amino acids 290-314 | pcDNA3.1-HA-  *Clu*_290-314_ |
| R7 | 5′-***TT***GGCCGCCTCGGCCTCAGGAACAGTCCACAGACAAGA -3′ | *SfiI* |  |  |
| F8 | 5′-***TT***GGCCATTACGGCCATGACCAACAACCCCTCCCAGGC -3′ | *SfiI* | Clu ORF spanning amino acids 315-381 | pcDNA3.1-HA-  *Clu*_315-381_ |
| R8 | 5′-***TT***GGCCGCCTCGGCCTCACTGGTCTTCGCCTTGCGTGA -3′ | *SfiI* |  |  |
| F9 | 5′-***TT***GGCCATTACGGCCATGTACTATCTGCGGGTCACCACGGTG-3′ | *SfiI* | Clu ORF spanning amino acids 382-460 | pcDNA3.1-HA-  *Clu*_382-460_ |
| R9 | 5′-***TT***GGCCGCCTCGGCCTCACTCCTCCCGGTGCTTTTTGCGG -3′ | *SfiI* |  |  |
| F10 | 5′-***TT***GGCCATTACGGCCATGGGCGTTTACGTGTGTGCC-3′ | *SfiI* | SelR ORF spanning amino acids 19-82 | pCMV-Myc-*SelR_19-82_* (Myc-tagged *SelR_19-82_*) |
| R10 | 5′-***TT***GGCCGCCTCGGCCTTAGAACTCGTGGCCCAACC-3′ | *SfiI* |  |  |
| F11 | 5′-***G***GAATTCATGTCGTTCTGCAGC -3′ | *EcoRI* | SelR ORF fused His-tagged and creating the mutation site from TGA to TGC | pET28(a)-*SelR′* (His-tagged *SelR′*) |
| R11 | 5′-*CG*GGATCCCTAGTGACCCTGGG -3′ | *BamHI* |  |  |
| F12 | 5′-***CG****GGATCC*CCACCAACAACCCCTCCCAG-3′ | *BamHI* | Clu ORF spanning amino acids 315-381 fused GST-tagged | pGEX-5X-1-*Clu_315-381_*(GST-tagged *Clu_315-381_*) |
| R12 | 5′-***CG****GAATTC*TTACTGGTCTTCGCCTTGCGTGAGG-3′ | *EcoRI* |  |  |
| F13 | 5′-***CG****GGATCC*ATGCGCAGCTTGATGCCCTTCTC-3′ | *BamHI* | Clu ORF spanning amino acids 227-449 fused His-tagged | pGBTNT-*Clu_α_* (His-tagged *Clu_α_*) |
| R13 | 5′-***CCG****CTCGAG*CTCCTCCCGGTGCTTTTTGCG-3′ | *XhoI* |  |  |
