# Supplementary material for: Direct Interaction of Selenoprotein R with Clusterin and Its Possible Role in Alzheimer’s Disease
Source: PLoS One. 2013 Jun 21;8(6):e66384. doi: 10.1371/journal.pone.0066384 (PMC3689823; doi:10.1371/journal.pone.0066384)
Supplement: Table S2 — Information on the images acquired through the sensitized-emission method. (DOCX) [file pone.0066384.s002.docx]

| Entry | Cells transfected with the following plasmid | Excitation wavelength /nm | Channel |
| --- | --- | --- | --- |
| Fig. A_1_/B_1_ | pECFP-C1/pECFP-C1-*SelR′* | 405 | CFP |
| Fig. A_2_/B_2_ |  | 405 | YFP |
| Fig. A_3_/B_3_ | pEYFP-C1/pEYFP-C1-*Clu* | 405 | YFP |
| Fig. A_4_/B_4_ |  | 515 | YFP |
| Fig. A_5_/B_5_ | pECFP-C1, pEYFP-C1/ pECFP-C1-*SelR′,* and pEYFP-C1-*Clu* | 405 | CFP |
| Fig. A_6_/B_6_ |  | 405 | YFP |
| Fig. A_7_/B_7_ |  | 515 | YFP |
