# Supplementary material for: Direct Interaction of Selenoprotein R with Clusterin and Its Possible Role in Alzheimer’s Disease
Source: PLoS One. 2013 Jun 21;8(6):e66384. doi: 10.1371/journal.pone.0066384 (PMC3689823; doi:10.1371/journal.pone.0066384)
Supplement: Method S1 — Confirmation of positive interaction in yeast. (DOCX) [file pone.0066384.s003.docx]

Supporting Method 1: Confirmation of positive interaction in yeast

Positive yeast colonies from library screening were selected from the quadruple dropout solid medium SD/-Ade/-His/-Leu/-Trp containing X-α-Gal and Aba. Plasmids extracted from the yeast were transformed into *E. coli* Top10 cells. Prey plasmid was screened with LB medium containing ampicillin and extracted from *E. coli* cells. The obtained prey plasmid and the NpGBKT7-*SelR′* bait plasmid were co-transformed into the Y2HGold yeast cells and grew on the SD /-Leu /-Trp /x-α-gal /Aba solid medium to check if the colonies turned blue. The prey plasmid was then sequenced and analyzed *via* bioinformatics.
