# Supplementary material for: Direct Interaction of Selenoprotein R with Clusterin and Its Possible Role in Alzheimer’s Disease
Source: PLoS One. 2013 Jun 21;8(6):e66384. doi: 10.1371/journal.pone.0066384 (PMC3689823; doi:10.1371/journal.pone.0066384)
Supplement: Method S2 — Protein expression and purification. (DOCX) [file pone.0066384.s004.docx]

Supporting Method 2: Protein expression and purification

For in vitro expression, the coding sequences of *SelR′*, *Clu_315-381,_* and *Clu_α_*were cloned into pET28(a), pGEX-5X-1, and pGBTNT expression vectors, respectively, before they were transferred to *E. coli* strain BL21:DE3 by heat shock, and one colony of each clone was cultivated at 37°C in 200 mL LB/kanamycin or ampicillin until an OD_600_ of approximately 0.5, when expression was induced by 1 mM isopropyl-beta-D-thiogalactopyranoside (IPTG). Six hours post-induction, the bacteria were collected by centrifugation (5,000 × *g* for 10 min). Total protein extracts were obtained by resuspension in lysis buffer (50 mM Tris-HCl, 100 mM NaCl, 2 mM EDTA, pH 8 for pET28(a)-*SelR′* and pGBTNT-*Clu_α_*; 1× phosphate-buffered saline (PBS) for pGEX-5X-1-*Clu_315-381_*), lysozyme treatment, and sonication. Lysed cells were centrifuged (13,000 × *g* for 10 min) at 4°C to remove insoluble material, and cleared supernatants were used to purify fusion protein by affinity chromatography using His and GST spintrap purification modules according to the manufacturer′s instructions (GE Healthcare).
