# Supplementary material for: Direct Interaction of Selenoprotein R with Clusterin and Its Possible Role in Alzheimer’s Disease
Source: PLoS One. 2013 Jun 21;8(6):e66384. doi: 10.1371/journal.pone.0066384 (PMC3689823; doi:10.1371/journal.pone.0066384)
Supplement: Method S3 — FRET analyses. (DOCX) [file pone.0066384.s005.docx]

Supporting Method 3: FRET analyses

For the sensitized emission assay, pECFP-C1-*SelR′*/pECFP-C1, pEYFP-C1-*Clu*/pEYFP-C1, or pECFP-C1-*SelR′* & pEYFP-C1-*Clu*/pECFP-C1& pEYFP-C1 plasmids were transfected into HEK293T, and images were acquired using an Olympus Fluoview FV1000 confocal microscope under the conditions listed in Supplementary Table S2. The CFP donor channel was acquired using donor excitation (λ = 405 nm) and the donor filter set. The acceptor channel (YFP) was acquired using acceptor excitation (λ = 515 nm) and the acceptor filter set. FRET was acquired using excitation (λ = 405nm) and the FRET filter set. Images were taken from donor, acceptor, and FRET samples using the same acquisition parameters. Donor and acceptor images were used to evaluate signal cross-talk caused by image settings and fluorophore properties. The acquired data was analysed using Olympus Fluoview FV1000 Toolbox software.

For the acceptor photobleaching assay, pECFP-C1-*SelR′* & pEYFP-C1-*Clu* plamids were transfected into HEK293T, and imaging was performed with a confocal lasr scanning mircroscope Olympus Fluoview FV1000, 60× oil immersion objective, using the acceptor photobleaching module. The acceptor signal was bleached in defined regions of interest (ROI) with 515 nm light at 98% laser power for 60 s. The change in donor (ECFP) fluorescence induced by acceptor photobleaching was quantified by comparing prebleach and postbleach images obtained by excitation at 405 nm. The acquired data was also analysed using Olympus Fluoview FV1000 Toolbox software. FRET efficiency was calculated as 1-Iprebleaching/Ipostbleaching, where Iprebleaching is the intensity of ECFP before the bleach in defined ROIs. Distance (r) was calculated as R_0_(1/E-1)^1/6^, R_0_ vablue for the pairing of CFP/YFP is 5.2767 nm. The background was determined by outlining a ROI in a region containing no cells. This background value was substracted from each value obtained in the cells. The mean FRET intensities obtained in at least 20 ROIs from at least three different transfections were measured for each protein pair. As controls, FRET in cells transfected with the tags alone, that is pECFP-C1 and pEYFP-C1 was also studied.
