# Supplementary material for: Direct Interaction of Selenoprotein R with Clusterin and Its Possible Role in Alzheimer’s Disease
Source: PLoS One. 2013 Jun 21;8(6):e66384. doi: 10.1371/journal.pone.0066384 (PMC3689823; doi:10.1371/journal.pone.0066384)
Supplement: Method S4 — Determination of methionine sulfoxide reductase activity. (DOCX) [file pone.0066384.s006.docx]

Supporting Method 4: Determination of methionine sulfoxide reductase activity

The reaction mixture (100μL) contained 100 mM Tris-HCl (pH 7.4), 15 mM DTT, 20 nM dabsylated methionine sulfoxide epimer, and 100 μg total protein samples. The reaction was carried out at 37°C for 30 min and stopped by adding 300 μL acetonitrile. After centrifugation at 12,000 rpm for 15 min, 20 μl of supernatant was injected onto a 4.6 × 75-mm C18 column pre-equilibrated with 55% 50 mM sodium acetate buffer, pH 4.73 and 45% acetonitrile. The column was developed using a buffer comprised of 55% 50 mM sodium acetate buffer, pH 4.73 and 45% acetonitrile, and the dabsyl derivatives were monitored by absorbance at 436 nm.
