# Supplementary material for: Direct Interaction of Selenoprotein R with Clusterin and Its Possible Role in Alzheimer’s Disease
Source: PLoS One. 2013 Jun 21;8(6):e66384. doi: 10.1371/journal.pone.0066384 (PMC3689823; doi:10.1371/journal.pone.0066384)
Supplement: Method S5 — Immunofluorescence assay. (DOCX) [file pone.0066384.s007.docx]

Supporting Method 5: Immunofluorescence assay

Cells transfected with Myc empty vector or Myc-tagged SelR′ were fixed with 4% formaldehyde in PBS for 10 mins, washed three times, permeabilized with 0.25% Triton X-100 in PBS for 10 minutes, washed, blocked with 5% bovine serum albumin-PBS for 1 h, then incubated for 1 hour with rabbit polyclonal Clu antibody (1:200) at room temperature. After three washes with PBS, cells were incubated for 1 h at room temperature with fluorescein isothiocyanate-conjugated goat anti-rabbit IgG. After further washes with PBS, nuclei were stained with 4’, 6-diamidino-2-phenylindole (1 μg·ml^-1^) and imaged by laser confocal microscopy.
